# Impact of HBV serological status on HIV virological efficacy of two‐drug antiretroviral regimens: A retrospective observational study on virologically suppressed people with HIV switching to lamivudine/dolutegravir

**DOI:** 10.1111/hiv.13765

**Published:** 2025-02-05

**Authors:** Pierluigi Francesco Salvo, Arturo Ciccullo, Elena Visconti, Francesca Lombardi, Carlo Torti, Simona Di Giambenedetto, Gianmaria Baldin

**Affiliations:** ^1^ Dipartimento di Sicurezza e Bioetica – Sezione di Malattie Infettive Università Cattolica del Sacro Cuore Rome Italy; ^2^ Infectious Diseases Unit, San Salvatore Hospital L'Aquila Italy; ^3^ Dipartimento di Scienze Mediche e Chirurgiche, UOC Malattie Infettive Fondazione Policlinico Universitario “Agostino Gemelli”, IRCCS Rome Italy

**Keywords:** antiretroviral therapy, HBV, HIV

## Abstract

**Objectives:**

This study aimed to evaluate the HIV virological efficacy of two‐drug regimens (2DR) with lamivudine (3TC) and dolutegravir (DTG) in people with HIV (PWH), classified by their hepatitis B virus (HBV) serological status. Specifically, it explored whether isolated anti‐hepatitis B core (anti‐HBc) positivity impacts virological outcomes.

**Methods:**

A retrospective observational study was conducted at Fondazione Policlinico Universitario Agostino Gemelli IRCCS, enrolling 606 virologically suppressed (HIV‐RNA < 50 copies/mL) PWH who switched to a 2DR regimen with 3TC/DTG. Participants were categorized into four groups based on their HBV serological status: hepatitis B surface antibody (HBsAb)‐positive/hepatitis B core antibody (HBcAb)‐positive, HBsAb‐negative/HBcAb‐negative, HBsAb‐positive/HBcAb‐negative, and isolated HBcAb positivity. Viral failure (VF) was defined as two consecutive HIV viral loads above 50 copies/mL or a single HIV viral load above 1000 copies/mL, and viral blips (VBs) as a single HIV‐RNA measurement between 50 and 200 copies/mL followed by suppression.

**Results:**

During 2216.4 patient‐years of follow‐up (PYFU), we observed 30 VFs (1.3 per 100 PYFU) and 63 VBs (2.9 per 100 PYFU). No statistically significant differences in VF or VB were noted between the serological groups. Additionally, no significant alanine aminotransferase (ALT) flares or HBV‐DNA breakthroughs were observed, with HBV‐DNA remaining undetectable throughout.

**Conclusions:**

The study supports the virological efficacy of 3TC/DTG‐based 2DR in PWH, regardless of HBV serological status. Isolated anti‐HBc positivity did not influence virological outcomes independently. Larger studies are warranted to further investigate HIV–HBV interactions in this context.

Research advancements on HIV treatment have led to the approval of two‐drug antiretroviral regimens (2DR) with lamivudine (3TC) and dolutegravir (DTG) for both treatment‐naïve and ‐experienced people with HIV (PWH). However, these regimens are not recommended for individuals who are hepatitis B surface antigen (HBsAg)‐positive, as 3TC, while having activity against HBV, is insufficient as monotherapy, requiring regimens with more sustained activity against HBV [1, 2]. An important clinical challenge arises in individuals with isolated anti‐hepatitis B core antibodies (HBcAb), a common HBV serological pattern observed in immunocompromised individuals [3], characterized by the absence of antigen (HBsAg) and antibodies (HBsAb), alongside positive HBcAb.

While isolated HBcAb positivity is not an absolute contraindication for 3TC/DTG, recent studies, such as that by Malagnino et al., have shown that HIV/HBV coinfection can impact the risk of HIV viral rebound after achieving viral suppression, particularly in individuals with HBsAg‐positive or HBcAb‐positive serology. Viral rebound in this study was defined as the occurrence of two consecutive HIV‐RNA values above 50 copies/mL after achieving viral suppression, typically defined as HIV‐RNA < 50 copies/mL. In a cohort of 5657 PWH, HBV coinfection was found to be associated with an increased risk of experiencing viral rebound after the initiation of antiretroviral therapy (ART), with those who were HBcAb‐positive showing a higher risk compared with HIV‐monoinfected individuals [adjusted hazard ratio (aHR) = 1.25, 95% confidence interval (CI): 1.00–1.55, *p* = 0.047]. These findings suggest that HBV infection, even in its inactive or occult form, may play a role in the stability of HIV suppression under ART [4].

The 3TC/DTG regimen, while effective in HIV suppression, is known to have a limited anti‐HBV activity. Although 3TC does provide some efficacy in controlling HBV, its use in coinfected individuals may not lead to a long‐term suppression of HBV replication, potentially leading to HBV rebound [2]. This limited activity, particularly in individuals with isolated HBcAb positivity, could compromise the stability of both HIV and HBV suppression, contributing to virological instability over time.

We conducted a retrospective observational study at the Fondazione Policlinico Universitario Agostino Gemelli IRCCS in Rome, enrolling virologically suppressed PWH (defined as HIV‐RNA < 50 copies/mL) for at least 6 months who switched to a 2DR regimen with 3TC/DTG.

Data analysed in the present study were selected from an internal observational database, which collects main clinical and demographic characteristics of every patient who gave written informed consent to personal data records since the time of HIV diagnosis. The creation of the database was approved by the “Fondazione Policlinico Gemelli” Ethics Committee (protocol number: 10978/15).

Our objective was to evaluate the HIV virological efficacy of 3TC/DTG by classifying participants based on their HBV serological status. HBV serological status, including both HBcAb and HBsAb results, was determined using tests conducted within 6 months prior to switching to 3TC/DTG. Participants were categorized into four groups based on their most recent results from this 6‐month period: HBsAb‐positive/HBcAb‐positive, HBsAb‐negative/HBcAb‐negative, HBsAb‐positive/HBcAb‐negative, and HBsAb‐negative/HBcAb‐positive. Participants with a history of both positive and negative HBV serology results were included based on their most recent results within this 6‐month time‐frame.

Criteria for eligibility included patient consent, age ≥18 years, viral suppression (HIV‐RNA < 50 copies/mL) at baseline, and a consistently negative HBsAg status, with the most recent HBsAg determination obtained within the 6 months prior to the switch to 3TC/DTG. Patients with any past positive HBsAg results or whose most recent HBsAg result was not negative within this time‐frame were excluded from the study.

Viroimmunological data of HIV infection were collected at the time of switch (baseline) and during ongoing follow‐up. For those who discontinued the 3TC/DTG regimen, data were collected until the date of discontinuation, while for those who continued the regimen, data were censored at the last available laboratory determination.

Viral failure (VF) was defined as two consecutive HIV viral load determinations above 50 copies/mL or a single HIV viral load above 1000 copies/mL. Viral blips (VBs) were defined as a single HIV‐RNA measurement between 50 and 200 copies/mL followed by a measurement below 50 copies/mL.

We enrolled 606 PLWH in our study. The distribution of participants was as follows: 260 in the HBsAb‐negative/HBcAb‐negative group, 180 in the HBcAb‐negative/HBsAb‐positive group, 126 in the HBcAb‐positive/HBsAb‐positive group, and 40 with isolated HBcAb positivity. The majority of participants were male (432/606; 71.3%) with a median age of 51 years (interquartile range: 43–58). Most were of Caucasian ethnicity (519/606; 85.6%), followed by African American (31/606; 5.1%) and Latin American (29/606; 4.8%) individuals. The main risk factors for HIV/HBV acquisition were sexual, with 42.6% of the population (258/606) being heterosexual and 40.9% (248/606) being men who have sex with men. A further 7.3% of participants (44/606) were people who inject drugs, and these were more prevalent in the subgroup with isolated HBcAb positivity [16/40 (40.0%) vs. 28/566 (4.9%) of other HBV serostatuses as a single group; *p* < 0.001]. Additionally, 12.0% of participants (73/606) showed serological evidence of past HCV acquisition and they were more prevalent in the group with isolated HBcAb positivity [17/40 (42.5%) vs. 56/566 (9.9%) of other HBV serostatuses as a single group, *p* < 0.001]. Regarding antiretroviral regimens prior to the switch to 3TC/DTG, 41.4% (251/606) were receiving integrase inhibitor‐based three‐drug regimens (3DR), 27.4% were on nonnucleoside reverse transcriptase inhibitor‐based 3DR, and 4.5% on protease inhibitor (PI)‐based 3DR, while 21.8% were on PI‐based 2DR. A substantial proportion (305/606; 50.3%) of participants switched to 3TC/DTG from an antiretroviral regimen containing tenofovir alafenamide or tenofovir disoproxil fumarate. The main reasons for switching to 3TC/DTG were simplification (64.7%), management of dyslipidaemia (10.6%) and addressing other toxicities or clinical needs (13.0%).

During 2216.4 patient‐years of follow‐up (PYFU), in the entire population we observed 30 VFs, at a rate of 1.3 VFs per 100 PYFU. The distribution of HIV VFs in the subgroups was as follows: seven in the HBcAb‐positive/HBsAb‐positive group (1.4 VFs per 100 PYFU), seven in the HBcAb‐negative/HBsAb‐positive group (1.1 VFs per 100 PYFU), 14 in the HBsAb‐negative/HBcAb‐negative group (1.5 VFs per 100 PYFU), and two in the isolated HBcAb positivity group (1.3 VFs per 100 PYFU).

Regarding VBs, during 2175.9 PYFU we observed 63 VBs with a rate of 2.9 VBs per 100 PYFU. The distribution of VBs in the subgroups was as follows: 13 in the HBcAb‐positive/HBsAb‐positive group (2.6 VBs per 100 PYFU), 17 in the HBcAb‐negative/HBsAb‐positive group (2.7 VBs per 100 PYFU), 29 in the HBsAb‐negative/HBcAb‐negative group (3.2 VBs per 100 PYFU), and four in the isolated HBcAb positivity group (2.7 VBs per 100 PYFU).

Survival analysis was conducted to assess the probability of remaining free from HIV virological failure (Figure 1a,c) and HIV viral blips (Figure 1b,d) in four separate comparisons: first, between the HBcAb‐positive/HBsAb‐negative group and all other serostatus groups (Figure 1a,b), and second, among the four groups themselves (Figure 1c,d). In each comparison, no statistically significant differences were observed.

**FIGURE 1:**
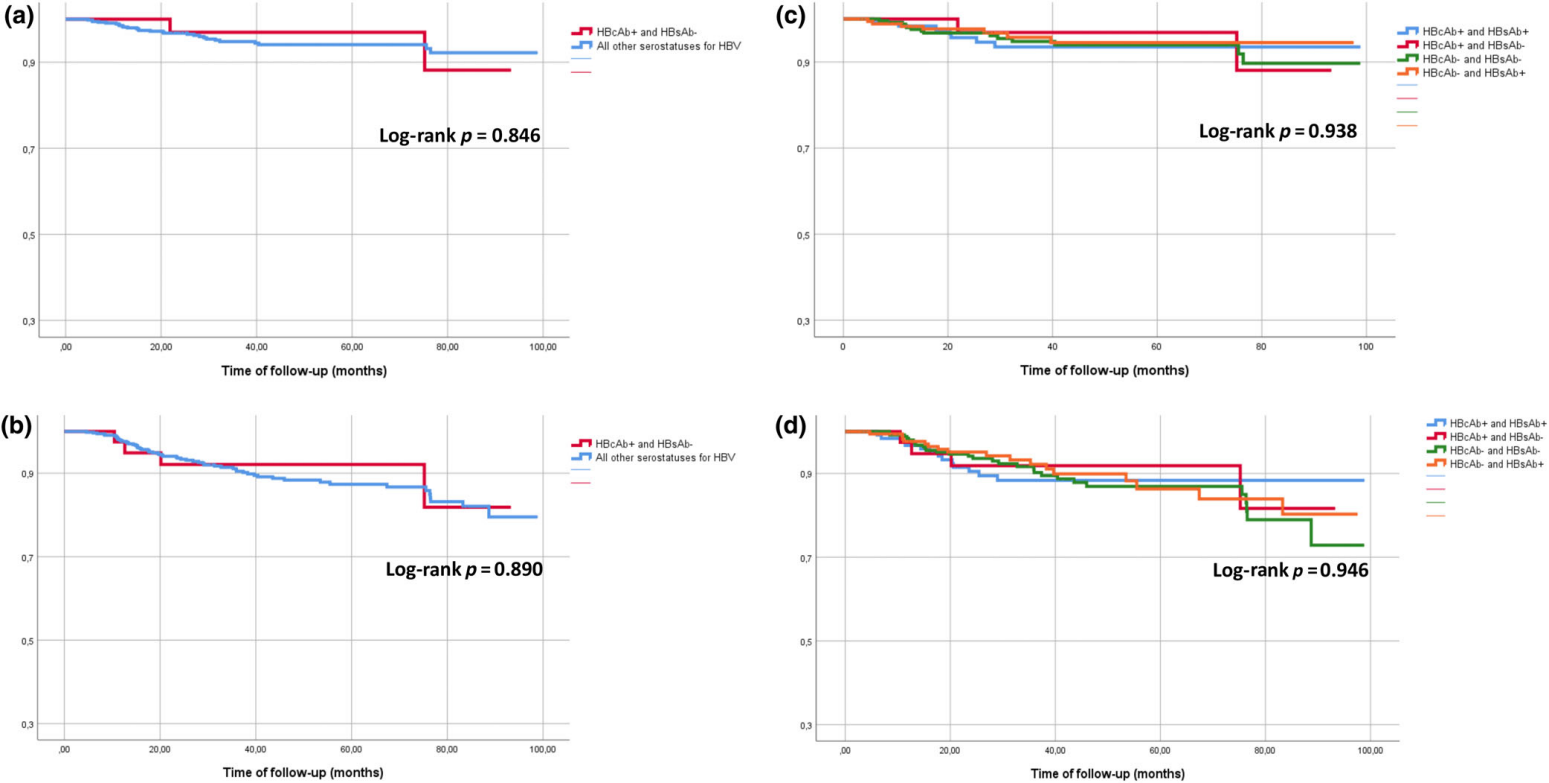
Probability of remaining free from HIV virological failure (a, c) and from HIV viral blips (b, d). Panels (a) and (b) represent the comparison between the hepatitis B core antibody‐positive (HBcAb+)/hepatitis B surface antibody‐negative (HBsAb‐) group and all other HBV serostatus groups considered as one. Panels (c) and (d) represent the comparison among the four groups.

During the follow‐up period, we analysed HBV‐DNA and ALT levels in the groups of patients with positive HBV serology (HBsAb‐negative/HBcAb‐positive and HBsAb‐positive/HBcAb‐positive). We observed that HBV‐DNA remained undetectable throughout the entire follow‐up period, with no ALT flares occurring, and no correlation was found between variations in ALT levels or HBV‐DNA status and HIV viraemia outcomes.

Our study has some limitations that should be taken into account when interpreting the data. One of the main limitations is the absence of HBV‐DNA measurements for the entire enrolled population. Unfortunately, these data were not available for our cohort, and this lack of information should be considered when interpreting our findings regarding the potential impact of HBV serology on HIV viral rebound. Additionally, we did not assess whether the patients who were HBcAb‐negative/HBsAb‐positive had been vaccinated against HBV at any point in their lives. While this information could provide further insights into the potential influence of prior vaccination on the risk of HBV/HIV‐related outcomes, we were unable to confirm the vaccination status of these individuals in our cohort.

These limitations should be considered when interpreting the results of our study and highlight areas for further investigation.

Despite these limitations, our data align with the existing literature from randomized clinical trials and real‐world clinical data, confirming the virological efficacy of 2DR strategies with 3TC and DTG in experienced PWH [5, 6].

However, contrary to some data reported in the recent literature, in our population of experienced PWH, HBV serological status does not appear to independently influence HIV virological outcomes, including the occurrence of HIV virological failure and HIV VBs. Furthermore, the fact that the individuals enrolled in our study are receiving a 3TC‐based regimen, which exhibits limited activity against HBV, does not appear to contribute to an increased risk of HBV viral rebound. Therefore, in our setting, 2DR strategies with 3TC/DTG seem to be effective in HIV viral suppression, even in PWH with HBcAb positivity.

However, further studies with larger sample sizes are warranted to confirm these results and explore potential interactions between HIV and HBV in this context.

## AUTHOR CONTRIBUTIONS

Manuscript conceptualization: P.F.S., A.C., G.B. Data collection: P.F.S., E.V. Data analysis: P.F.S., F.L., G.B. Methodology: P.F.S., F.L., C.T., S.D.G. and G.B. Drafting the manuscript: P.F.S. Manuscript editing: A.C., E.V., F.L., C.T., S.D.G., G.B. All authors have read and agreed to the published version of the manuscript.

## FUNDING INFORMATION

This work was supported by internal funding.

## CONFLICT OF INTEREST STATEMENT

AC received support for travel to meetings from ViiV Healthcare. SDG was a paid consultant or member of advisory boards for Gilead Sciences, ViiV Healthcare, Janssen‐Cilag, MSD and Bristol‐Myers Squibb. All other authors have no conflicts to declare.
